# The impact, perceptions and needs of parents of children with epidermolysis bullosa

**DOI:** 10.4102/safp.v66i1.5897

**Published:** 2024-06-06

**Authors:** Antoinette V. Chateau, David Blackbeard, Colleen Aldous, Ncoza Dlova, Cassidy-Mae Shaw

**Affiliations:** 1 Department of Dermatology, Faculty of Health Sciences, Greys Hospital, Pietermaritzburg, South Africa; 2 Department of Dermatology, Faculty of Health Sciences, University of KwaZulu-Natal, Durban, South Africa; 3 Department of Psychology, Faculty of Health Science, Greys Hospital, Pietermaritzburg, South Africa; 4 Department of Psychology, Faculty of Health Science, University of KwaZulu-Natal, Durban, South Africa; 5 School of Clinical Medicine, Faculty of Health Sciences, University of KwaZulu-Natal, Durban, South Africa

**Keywords:** epidermolysis bullosa, parents, interpretative phenomenological analysis, impact, perceptions, needs

## Abstract

**Background:**

Epidermolysis bullosa (EB) is a rare, incurable genodermatosis that presents with blistering and skin fragility. Complications can be localised or generalised, limited to the skin or have systemic effects resulting in death. Caring for a child with this painful condition can have a profound effect on the quality of life of parents and the family. There is currently no published research on the lived experience of parents caring for a child with EB in a resource-limited environment in Africa.

**Method:**

This qualitative research used interpretative phenomenological analysis with the aim of understanding the lived experiences of parents caring for children with EB. Semi-structured interviews were conducted with 13 participants between May 2022 and October 2023. Guba’s framework of trustworthiness was used to ensure rigour.

**Results:**

Seven experiential themes with associated sub-themes were identified. The themes were (1) grappling with understanding EB, (2) the psychological experience, (3) living with the responsibility, (4) barriers to feeling supported, (5) changing relational dynamics, (6) experience of healthcare professionals and (7) parental needs.

**Conclusion:**

Parents caring for children with EB face emotional, physical, psychosocial and financial challenges. Addressing parents’ needs and concerns will go a long way in decreasing this burden. A biopsychosocial approach with an awareness of cultural context is essential for family-centred holistic EB care.

**Contribution:**

This is the first study in Africa that focussed on the lived experiences of parents caring for a child with EB.

## Introduction

Epidermolysis bullosa (EB) is a rare genetic skin condition classified into four main groups with over 30 subtypes varying in clinical presentation that is characterised by extreme skin fragility because of genetic mutations in the proteins that maintain the integrity of the skin.^1,2^ There is a paucity of information on specific aspects of the disease, consequently some of the data referenced are older than 5 years. The prevalence of EB varies per country with 11.1 per million noted in the United States (US).^3^ There is no EB registry in South Africa and hence the prevalence of EB in South Africa and KwaZulu-Natal is not known.

Patients may present with blister formation, erosions and granulation tissue involving skin, mucosa, hair, teeth and nails.^4^ Complications include potentially fatal infections, failure to thrive, pubertal delay, gastrointestinal, ocular, musculoskeletal, squamous cell carcinoma and psychological sequelae.^5^ There is no cure to date, and treatment is aimed at preventing blister formation and wound care, which can be financially burdensome.

In a resource-limited environment such as South Africa, with an already strained health system, little attention is given to rare diseases,^6^ including EB. Accurate diagnosis of EB is often delayed because of a lack of diagnostic techniques. In addition, many essential resources, such as wound dressings, are not available in the government sector and are only partially funded by private medical aids.

Caring for a child with EB is life-changing and places large demands on parents. Parents caring for children with chronic illnesses are impacted in the areas of health, interpersonal relationships, careers and finances, and emotional and mental well-being.^7^

In understanding parents’ experiences of caring for children with EB, it is important for healthcare practitioners (HCPs) to appreciate the cultural background, beliefs and practices of patients and their caregivers to fully engage with the patient and family. An open dialogue assists in compliance and openness to discuss treatment practices that may be harmful or result in the progression of the disease, such as the application of untested herbal mixtures or scarifications to open lesions in patients with EB. A study by Ayibor showed that 57% of patients who visited traditional healers had scarification into which black burnt herbal preparations were rubbed.^8^

A comprehensive biopsychosocial approach is essential for the holistic care of the patient and their family while being cognisant of the associated difficulties, challenges, cultural perspectives, and financial limitations.

## Research methods and design

### Study design

Grounded in a qualitative research framework, the study used interpretative phenomenological analysis (IPA)^9^ to describe and interpret the lived experiences of parents of children with EB. The researcher embraced a hermeneutic epistemology that recognises the freedom of human beings in examining their world within context and that knowledge is derived from studying first-person experiences.^9^ The principal investigator’s (PI) interpretative framework is influenced by previous research in EB,^10,11,12^ experience from specialist medical practice in the area of EB and professional relationships with parents of patients with EB that have developed over time during the treatment of patients. Understanding that a researcher cannot be completely objective, the PI undertook collaborative reflection and regular introspection and included a collaborator (CS) in the analysis process to ensure that subjective feelings and preconceptions were not interfering with or influencing the research process.

### Setting

Interviews were conducted in private offices at Grey’s Hospital in Pietermaritzburg and Victoria Mxenge Hospital in Durban in spaces removed from clinical settings to ensure privacy, confidentiality and comfort with six parents, while another six were interviewed via Zoom and the final one via telephone.

### Study population and sampling strategy

Thirteen parents participated in the study (Table 1) based on the primary inclusion criterion of being a parent of at least one child with EB. Owing to the very rare nature of EB, purposive sampling was used to access participants who would be able to provide rich information on the phenomenon of study.^9,13^ Seven participants were known patients of Grey’s Hospital and Victoria Mxenge Hospital Dermatology departments. The remaining six participants were accessed through DEBRA (Dystrophic EB Research Association) in South Africa. Respondents 6 and 7 were re-interviewed after the loss of their second child to EB.

**TABLE 1:** Parent demographics.

Respondent number	Gender	Race	Employment status	Age of child	Relation to other participants
1	F	A	U	5 years	-
2	F	A	U	18 years	-
3	F	A	U	1 month	-
4	F	A	U	2 months	-
5	F	A	S	2 months	-
6	F	A	U	3 months (passed away during the study period)	Partner to 7
7	M	A	E	3 months as above	Partner to 6
8	F	W	E	30 years	-
9	F	I	E	37 years	Partner to 10
10	M	I	E	37 years as above	Partner to 9
11	F	W	E	13 years	-
12	F	M	E	5 years	Partner to 13
13	M	I	E	5 years as above	Partner to 12

F, female; M, male; A, African people; W, white people; I, Indian people; M, mixed race people; U, unemployed; E, employed; S, student.

### Data collection

Semi-structured interviews were conducted between May 2022 and October 2023 by a postgraduate psychology student trained in the specified interviewing method and oriented to the study context by the PI and co-author, D.B. Questions were tested in a pilot phase (Appendix 1), and a final set of semi-structured open-ended questions was drawn up for the research interviews. No changes were made to the interview schedule after the pilot phase. IsiZulu was the predominant language spoken by the study population. Privacy, confidentiality and comfort were considered for all interviews because of the sensitive nature of the topic, including being able to speak in their home language as the interviewer was fluent in both English and isiZulu. Interviews were digitally recorded or, if conducted virtually, using the Zoom^©^ record function. Interviews ranged from 80 min to 120 min. Debriefing took place between the interviewer and the PI after each interview to adapt questioning and allow the PI to begin preliminary familiarisation with the data. Interviews were transcribed and translated verbatim by the interviewer.

### Data analysis

Interview transcripts underwent systematic inductive analysis by the PI and a collaborating researcher (CS), initially independently and then following a collaborative process. The data were analysed according to the four-step process outlined by Smith and Nizza^14^: (1) Transcripts were carefully read and exploratory notes were made on each transcript; (2) the PI and CS formed independent experiential statements from their respective exploratory notes to capture the meaning of participants’ experiences; (3) the PI and CS engaged in research meetings to identify commonalities and reach consensus on interpretations and began clustering themes together, and continued rigorous dialogue to reach agreement on experiential themes that best-captured experiences; and (4) final experiential themes and sub-themes were compiled (Table 2).

**TABLE 2:** Experiential themes and sub-themes.

Experiential themes		Sub-themes
1.	Grappling with understanding EB	1.1. Difficulty understanding the pathophysiology 1.2. Seeking out explanations 1.3. Improved understanding from practical care education 1.4. Paradox of seeking normality yet recognising the necessity for differentiation
2.	The Psychological experience	2.1. Emotional toll 2.2. Fragility of life 2.3. The vulnerable parent
3.	Living with the responsibility	3.1. The unheard expert 3.2. Finding a means to cope 3.3. Sacrifices in quality of life
4.	Barriers to feeling supported	4.1. Family perspectives 4.2. Social judgement and stigma
5.	Changing relational dynamics	5.1. Attachment to the child with EB 5.2. Impact on parental unit relationships 5.3. Changing dynamics for other children
6.	Experience of healthcare professionals	6.1. Unhelpful healthcare professionals 6.2. Helpful healthcare professionals
7.	Parental needs	7.1. Need for specialist care centres and home based care 7.2. Financial assistance 7.3. Psychosocial support

EB, epidermolysis bullosa.

Sources of triangulation, including a reflective diary, research team correspondence and debriefing notes, were used to ensure a thorough, accurate analysis. Final integration occurred through reflection and review during the write-up.

Guba’s framework of trustworthiness,^15^ further refined by Shenton,^16^ was used to ensure rigour, focussing on credibility, transferability, dependability and confirmability. Investigator triangulation, research meetings and the maintenance of an audit trail account for credibility. Clear descriptions of the research context and methodology and rich descriptions of the phenomenon under study were presented, ensuring transferability. Having a third-party interviewer and a CS in the analysis process, thereby reducing the bias of the PI, achieved confirmability. All research activities were documented, and consensus among researchers on experiential themes was achieved, ensuring dependability.

### Ethical considerations

Ethical approval to conduct this study was obtained from the University of KwaZulu-Natal, Biomedical Research Ethics Committee (reference no.: BREC/00003768/2022). Hospital gatekeeper approval was obtained from the management of each institution. Written informed consent for participation and audio recording was obtained from participants in each participant’s preferred language (English and isiZulu) prior to each interview. All participant data were de-identified and coded with participant numbers to ensure the confidentiality and anonymity of participants. All information was stored in a password-protected format, with codes stored separately from the data. All copies of data and recordings are planned to be destroyed 5 years after the completion of the study. Commitment to sharing study findings with participants was honoured.

## Results

The experiential themes and sub-themes identified are presented in Table 2.

### Theme 1: Grappling with understanding epidermolysis bullosa

Epidermolysis bullosa is an extremely rare disease, and all participants were unaware of its existence prior to their children receiving the diagnosis.

#### Sub-theme 1.1: Difficulty understanding the pathophysiology

Parents had difficulty making sense of the disease, particularly when it came to understanding the aetiology. While the participants acknowledged that their HCP has explained that the condition is genetic, they struggled with accepting this explanation when their child was the first in their family to have this disease. Parents found it easier to attribute it to an allergic reaction, to an infection or to understand it in the context of ancestral beliefs:

‘When I first saw her, I told myself it was something that was going to disappear even when the doctors had told me what kind of disease it was. I was really hopeful that since I didn’t know such a thing and it has never happened in my family, it won’t start with me so I didn’t believe them at first that it was EB.’ (Respondent 5)

One parent could not comprehend any medical aetiology and viewed her child’s illness as a ‘gift from the creator’ (Respondent 2). Respondent 8 relayed that she was told by a geneticist treating her child that ‘he may have bunked the skin lecture where they discussed the condition because he’s never heard of it’.

#### Sub-theme 1.2: Seeking out explanations

Being driven by a need to understand the condition and left wanting when many HCPs did not know about EB either, the participants demonstrated a consistent effort to make sense and find explanations. Some parents were so determined to understand the disease at a time preceding the Internet and emails that they ‘took a piece of skin to [name of hospital] in London’ (Respondent 8) for a definitive diagnosis. Others travelled to centres within South Africa, self-referring to HCPs in search of answers, seeking out ‘a second or third opinion’ (Respondent 9) in the hope of a different diagnosis, prognosis and outcome. Respondent 5 acknowledged that the doctors were trying but they still ‘wanted to try in [*their*] own way maybe to try to go to other doctors’.

Parents sought answers in traditional and religious belief systems that were outside of their customs and religion, even at the expense of being rejected by their families.

Parent support groups and organisations such as DEBRA South Africa and Rare Diseases South Africa were significant sources of support to many parents. They also searched for information online, as voiced by Respondent 1: ‘I was going online Google to find out about EB’. Feeling informed empowered the parents and encouraged acceptance.

#### Sub-theme 1.3: Improved understanding from practical care education

Parents felt relieved and supported by receiving practical advice and information. Physically caring for their child gave them a sense of comfort, relief and empowerment, which counteracted their insecurity around understanding and lack of knowledge of the disease.

Additionally, conceptualising the disease appeared to be easier when parents were given a metaphor to understand the disease:

‘I understood it because they explained what causes it to me. The doctor made an example with bricks saying it’s like when you don’t mix them with cement then obviously they fall. The way she explained it is the way it was happening on the baby’s skin.’ (Respondent 5)

#### Sub-theme 1.4: Paradox of seeking normality yet recognising the necessity for differentiation

Parents experienced a pendulum swing between the poles of desperately wanting their child to be a ‘normal child’ (Respondent 1) and realising that their child had physical challenges, which were recognised by others and provided with the necessary assistance.

One parent simultaneously expressed wanting normality for her child and despondency that the South African Social Security Agency (SASSA) had declined a care dependency grant ‘more than four times’ (Respondent 1) on the apparent basis of them classifying her child as having ‘a normal sickness’ (Respondent 1).

### Theme 2: The psychological experience

Caring for a child with EB significantly affected the physical and mental well-being and psychological functioning of parents.

#### Sub-theme 2.1: Emotional toll

Parents experienced a plethora of emotions from the moment their child was born. The momentous occasion of birth was an ambivalent experience for most of the parents, with Respondent 4 succinctly capturing this as ‘I felt happy, but I also felt disappointed’. The roller coaster of emotions is never-ending for the participants.

A commonly shared experience among participants was the considerable burden experienced by the genetic nature of the disease and guilt and self-blame that they were responsible for passing on the condition to their children:

‘I saw it as my fault. Maybe I caused this, I blamed myself, and it was like everything that was happening, I caused it to happen.’ (Respondent 6)

Parents also experienced guilt for not being able to support the complex needs of their children. They associated the progression and outcome of the disease with their inability to provide essential baby supplies such as soft blankets and non-friction clothing.

Respondent 6 also presented with feelings of guilt related to feeling that she was ‘failing [*her partner*]’, having lost two babies to EB and a third, which was terminated at 4 months gestation years before.

There was a sense of helplessness in not knowing what to do to comfort their children and feeling shame at not being able to take away the pain and condition:

‘It hurt me seeing her in this condition. It brought me sorrow because she couldn’t tell me where it was hurting.’ (Respondent 5)

‘It was heart-breaking to watch an innocent child go through this pain. I felt that it should have been me rather than the child.’ (Respondent 9)

There was a common fear among parents that others did not know how to handle their children gently and thus may cause harm or not know what to do in an emergency. Thus, their children were not allowed to partake in activities such as sleepovers that their friends enjoyed. They also shared a fear of who would care for their children if they passed away.

A father shared that his ‘greatest fear was when I found out [*his daughter’s name*] was pregnant’ (Respondent 10), fearing that he could not handle seeing another child being born with EB go through this disease. Parents feared their children being bullied and ostracised, having observed others staring at their children and the concern and fear that their children would not make meaningful friendships or romantic relationships.

Regret was also shared among the respondents. ‘I have regrets that maybe I shouldn’t have had another baby because I face the same thing’ (Respondent 6). While he understood the desire for a child without EB, Respondent 7 felt resentment towards his partner for not being responsible with contraceptives, resulting in having to ‘relive their previous ordeal’.

Parents experienced ambivalent emotions around caring for their children. Respondent 7 expressed feeling ‘cursed’, going on to explain:

‘It’s just like having a disabled child. You see, you won’t be able to be happy even if you want because you look at her and tears fill your eyes because I would look at her feet; she didn’t have skin, the meat wasn’t there.’ (Respondent 7)

Ambivalent emotional experiences extended to death as well. Sadness and grief at the loss of their babies conflicted with relief that their baby was no longer suffering and was in pain:

‘Let’s release her because of the pain she was feeling, we should rest our hearts because she was sick in front of us.’ (Respondent 5)

#### Sub-theme 2.2: Fragility of life

Anticipatory grief was noted in all parents of children with EB. Participants were devastated and even traumatised at the diagnosis and prognosis shared by HCPs and then had to face the daily experience of constantly preparing for the inevitable death of their child:

‘Once she was past that six months where she should have died and she got to the age of one, I can’t remember being so frightened.’ (Respondent 8)

In this state of constant fearful anticipation, parents struggled to feel comfort or hope with thoughts such as ‘I usually think that they are just consoling me that she will be alright’. (Respondent 5)

#### Sub-theme 2.3: The vulnerable parent

Some parents were physically or emotionally abandoned by their partners and had to care for their children alone. A parent shared that her partner physically abused her during her pregnancy. Parents with insight self-referred to psychologists for assistance. A couple shared that they had turned to alcohol, pain medication and marijuana during difficult times but soon realised that these vices only numbed their emotions and did not help or change their reality:

‘I was smoking a lot of dagga. She would cry until she had a headache, I ended up buying pills for her, we always had a box of pills I would try talking to her or try to do something she ended up starting alcohol.’ (Respondent 7)

While others had the support of their partners, they had poor coping strategies. One mother’s mental health was severely impacted to the point of her attempting suicide as a result of feeling judged by her community for passing on the disease to her child (as the carrier of a genetic disorder) and her questioning her role in her baby’s illness:

‘Everyone was looking at me differently; it’s my fault that’s why the baby is like this. I was trying to figure out what I’m doing wrong, what I’m eating, what is wrong with me; maybe it’s me. I don’t know; I even tried to kill myself.’ (Respondent 6)

A husband has noted his concern regarding the negative effect and toll their baby’s illness has taken on his partner and the depth of anger, pain and trauma caused by the community, her family and the HCPs:

‘I am feeling concerned that I should have done something if I knew she was feeling like this because you can see she is angry, she has been traumatised, she is a dead person. She needs to get back alive; refresh the mind.’ (Respondent 7)

### Theme 3: Living with the responsibility

Having a child with EB is all-consuming, and parents fully experienced the burden of responsibility in this regard.

#### Sub-theme 3.1: The unheard expert

Parents have taken on the unwanted role of an expert, an HCP and an advocate for their children.

Surrounded by healthcare workers who are not *au fait* with the condition, participants had to watch their child’s condition deteriorate and physically care for their child themselves despite hospitalisation. Respondent 1 was told:

‘There is no use for you to be here cause you doing everything yourself, dressing yourself. The nurses they don’t know, so better to go do it at home that’s how bad it was.’

Respondent 12 experienced healthcare workers not knowing how to remove bandages and ‘[*ripping*] the whole skin off her legs’.

Parents have had to advocate for their children at times when they were at their most vulnerable and fight the medical insurance providers for their children to receive care. One participant detailed that:

‘[*Y*]ou have to continuously send motivations and information through to them, yet they know the type of condition if they have doctors sitting on the board when they authorising these things. They should be qualified and know the condition, so I escalated it because to me it’s discrimination, a disease is a disease.’ (Respondent 13)

#### Sub-theme 3.2: Finding a means to cope

Coping strategies varied across parents. Some felt utterly depleted with no coping mechanisms to deal with their child’s illness, while some found comfort in religion and the support of family and friends. ‘When I was coming there to the hospital, the brethren would call and just pray, that is what helped me to cope, there’s nothing else besides that’ (Respondent 5).

Socioeconomic factors revealed a stark disparity in coping strategies, with more affluent parents being able to employ helpers to care for their children while they have much-needed downtime or they took up yoga. In contrast, others had little or no support or rest:

‘Our helper has helped out in a period of our lives where there was a whole lot going on I think if we didn’t have her I might you know not have been a good wife and a good mom so yeah we did have [*name of person*]. I did go to the local homeopath and do some TRE^®^ [*Tension, Stress and Release*] classes and things like that just so I felt I had time for me and then I took up yoga a couple of years ago.’ (Respondent 11)

#### Sub-theme 3.3: Sacrifices in quality of life

Parents have been metaphorically wrapped up in caring for their children who are physically wrapped in bandages, illustrated through Respondent 5 stating, ‘Yah, I gave her everything and gave up everything’. Their time was consumed with caring for their children, with little time left for anything else, at the expense of their well-being, describing it as ‘exhausting’ (Respondent 12).

Mothers had given up their jobs or tertiary studies to care for their children, which has curbed career opportunities and greatly strained the family budget.

Parents and the other children in the family had given up going to functions with family and friends, with Respondent 13 saying, ‘I don’t have a social life anymore [*laughs*]. I don’t go out much.’ Parents experienced those around them as not being mindful of the family’s challenges and the difficulties around the inherent need to plan for socialising. This led to isolation, animosity and tension within the family.

### Theme 4: Barriers to feeling supported

Parents lacked support from family and society at large, which added to the challenges and trauma of caring for their children with EB.

#### Sub-theme 4.1: Family perspectives

Irrespective of their cultural background, parents felt accused by their family of being responsible for their children’s illness. Blame was a shared experience with Respondent 12 indicating, ‘They put the blame on me and that did hurt’, and Respondent 9 detailing how ‘there was a lot of mudslinging’, from her mother-in-law accusing her of her behaviour causing their child’s EB. There were also cultural associations to the acquisition of the disease; for example, ‘they were saying I have to do something for ancestors to say sorry to the ancestors’ (Respondent 7) and ‘it was never looked at as a medical condition, it was always that there were some other forces’ (Respondent 9), implying attribution of the cause of the illness to ancestral anger directed at what parents had done in the past.

Parents felt conflicted between their belief in the allopathic healthcare system and their traditional beliefs.

Family members blamed parents for the disease and failed to show support for the death of their child.

#### Sub-theme 4.2: Social judgement and stigma

Participants experienced significant social judgement, and many were perceived to have burned their babies. They described people in the community as ‘sceptical’ (Respondent 11) and described trauma around being investigated by authorities for suspicion of burning a child.

Parents shared the trauma of having to use public transport because of the stigma related to people thinking that EB is contagious and being questioned about the treatment of their children:

‘People don’t understand at all … they’ll ask me, did you burn your child. They judge me. Did you burn your child with water.’ (Respondent 1)

A family shared that they had felt so victimised by the community that they left behind their home and lucrative business in the area.

### Theme 5: Changing relational dynamics

The significant strain on relationships that caring for a child with EB has was evident in the participants.

#### Sub-theme 5.1: Attachment to the child with epidermolysis bullosa

All participants shared a deep bond with their children, expressing feeling more connected and even admitting to feeling ‘like I love her most than all these children; that’s how I feel maybe because of her condition’ (Respondent 1).

Parents recognised that the strengthened bond shared with their child also had some negative aspects:

‘I think the downfall there is because her condition, I’m not as strict on her as I was with the boys, like the boys will say to me you need to discipline her.’ (Respondent 12)

#### Sub-theme 5.2: Impact on parental unit relationships

Couples shared that caring for their children had considerably strained their relationship. Parents shared feeling tested by partners under circumstances of different and extreme caring requirements and resentment towards their partners for lack of caring capacity:

‘[*I*]t has affected us because sometimes he looks at me like someone who doesn’t do things properly.’ (Respondent 6)

‘[*S*]he is very careless, she is lazy, you would hear the child crying uncontrollably, and she would easily get angry; she would even bite her.’ (Respondent 7)

Given their caring responsibilities, little quality time is available for these parents. Some parents were abandoned by their partners or had partners who were emotionally detached and unsupportive. Most couples shared that their romantic relationships suffered a great deal. One mother quipped, ‘it’s almost like the romance is gone because there is another man.’ (Respondent 11)

While all participants experienced the strain, some couples bonded over being the only ones who fully understood the inherent challenges faced. ‘We kind of joined forces and decided to do this together and like I say we yeah we just had to manage what we were feeling’. (Respondent 10)

#### Sub-theme 5.3: Changing dynamics for other children

Parents were guilt-stricken that their time was consumed with taking care of the needs of their child with EB at the expense of their other children:

‘It has been hard particularly for my other daughter as well. I didn’t realise what was happening with my other daughter, it is because of the attention that I gave [*name of child with EB*] that apparently [*name of daughter*] needed to get out of the home, so she started travelling at a young age, and she stayed away from her family.’ (Respondent 8)

Some parents had been firm in expecting other children to step up and assist with the care of their siblings. Some of their children openly expressed dissatisfaction and its impact on them, while others seemed to understand the dynamic.

### Theme 6: Experience of healthcare professionals

A significant mediator of experiences for the participants was the approach, knowledge, perspectives and attitudes of the HCPs they dealt with, including nurses and doctors.

#### Sub-theme 6.1: Unhelpful healthcare professionals

Participants had difficult experiences with some HCPs who had an insensitive approach in sharing difficult news and were very clinical and factual about the fatality of the condition without providing any information, support or hope. The quote from Respondent 8 indicates how blunt some HCPs were in giving limited survival timeframes:

‘A doctor told me that my child was going to die … that I shouldn’t get close to her … she has the lethal form.’ (Respondent 8)

Healthcare practitioners’ poor knowledge was also evident:

‘They did not know what to do. Each time they tried to change her or dressing or do anything, the skin was just falling off. I remember the one doctor said that they read about it in a textbook, and they’d probably done it in med school, but they’ve never seen a case you know, dealt with anything like that, so in [*name of city*] for instance where [*name of child*] was born, there wasn’t anybody that had any experience with EB.’ (Respondent 9)

Parents felt dismissed when trying to share their concerns that certain techniques or dressings may cause further damage to their children’s skin and felt stigmatised by HCPs refusing to touch the patient or judging the parents:

‘The doctor, she never even touched my daughter; she’d say sit there, and she’s far like that she won’t even touch the child.’ (Respondent 1)

Some HCPs had a distorted perspective as to the cause of the condition and accused parents of harming their children:

‘When you are going to the clinic … the nurse don’t have any information about this disease, and she will ask you why did you burn the baby she will be harsh to you.’ (Respondent 6)

This, along with the perceived insensitivity of medical staff, was experienced as traumatising and reinforced the judgement from communities, leaving them feeling alone to the extent that they were willing to stop attending medical facilities entirely (Respondent 6) or fight their way in through security to see their child (Respondent 7):

‘They say I must terminate my baby, then after the termination and they took me to stay with people who just gave birth to the babies, so happy with their babies. I even run away. I didn’t even finish my medication. I was going to be like crazy.’ (Respondent 6)

‘Our baby was almost dying, and then when we go there, they didn’t want to help us. The securities were telling us to stand aside, and we even fight physically. The nurse said a man must not be in the ward; you don’t know the pain that I am feeling right now. I am not allowed. I have to sit outside by the bench in the night. I had to sleep outside.’ (Respondent 7)

#### Sub-theme 6.2: Helpful healthcare professionals

Where HCPs were empathetic, available and engaged with them, parents had much more positive experiences. Healthcare practitioners who were unfamiliar with EB but took the time to research the condition and include the parent in the child’s care to ensure that there was no further trauma caused were appreciated by participants and recognised as meaningful in their experience:

‘I have never felt like that since I came here … I always thank her. Since I had this child here, I have never felt so special like the way you do; you always support me, you always guide me, even if I have to come late; you don’t have like the way other doctors do, and they don’t have any understanding but you always go the extra mile.’ (Respondent 1)

Interestingly, parents felt more comfortable and had better experiences with their general practitioners (GPs) as well as primary health practitioners compared to their specialists. They felt that the former HCPs were empathetic, had established trust and rapport, and emotionally supported the parents. This was in comparison to their view of most specialists who had a more extensive knowledge base and experience of EB but were perceived as purely clinical:

‘[*Name of child*] found a fantastic GP not a dermatologist in [*name of city*], she was a very good GP, she’s fantastic, we just bounce a lot of things off her.’ (Respondent 8)

### Theme 7: Parental needs

#### Sub-theme 7.1: Need for specialist care centres and home based care

Many parents reported a delay in the diagnosis of the disease because ‘it felt like nobody knew what EB was’ (Respondent 8). Parents expressed a need for specialist centres to be established for expert care and rapid diagnosis, as well as for connection with other parents of children with EB.

Respondent 12 expressed a need for medical insurance to recognise the importance of and approve home-based care.

#### Sub-theme 7.2: Financial assistance

Caring for a child with EB carries substantial financial burden for families. Frequent hospital visits, the cost of medication and dressings not available or out of stock in the government sector or co-payments if parents had medical insurance appeared to place a considerable strain on the family:

‘I need money for transport and not that I have to borrow. I would know that if I have a baby’s appointment, maybe sometimes I end up not coming because I don’t have money, and for the baby to be alright and wear the required things.’ (Respondent 6)

This financial burden was compounded by the loss of income when mothers had to give up their jobs to care for their children. Some parents had been abandoned by their partners at the birth of their babies and had to rely on social grants and the generosity of other family members to survive. Many shared a need for financial support and assistance from the government to pay for transport costs to attend medical appointments, wound care and to buy suitable clothing:

‘Maybe if she can get a grant, I can look after her.’ (Respondent 3)

Some parents had been declined the care dependency grant by SASSA as the agency views this incurable and debilitating disease as not warranting special care. ‘[*doctors name*] also tried to put me in that ah you know that grant for sickness, they actually denied me more than four times’ (Respondent 1).

#### Sub-theme 7.3: Psychosocial support

The need for psychological support and counselling was evident, with parents indicating an awareness of the impact on their mental health and functioning and recognising the need for counselling. However, they were also unaware of what services exist or where or how to access them.

## Discussion

This study aimed to capture the lived experiences of parents caring for a child with a rare disease in a resource-limited environment. Seven themes were identified: parents grappling with understanding EB, their psychological experiences, living with the responsibility, barriers to feeling supported, changing relational dynamics, the experience of healthcare professionals and parental needs. The findings from the study that caring for a child with a rare, chronic and incurable condition such as EB places a significant amount of strain on parents and caregivers were consistent with previous observations by Martin et al.^17^

In this study population, consistent with many other studies,^18,19,20^ mothers were the primary caregivers and made up the majority of the participants,^10^ apart from one father who was highly invested in the day-to-day care of his child as his partner was not coping.

Three mothers had shared that they had been abandoned by their partners when their children were born, and one mother was estranged from her husband. It appeared that fathers played the more traditional role of breadwinners, did not attend doctors’ appointments, and were not involved with the actual care and dressing changes. Similar findings were noted by Kahraman et al. who showed that fathers play a less active role and were thus less affected than mothers in caring for their children.^7^ Some fathers admitted that they could not handle seeing their children in pain, and others physically or emotionally abandoned their partners. A study by Cardinali et al. noted that fathers focussed on providing for the family, were concerned about the future, actively sought out practical solutions and maintained a social life whereas mothers were the main caregivers, gave up their careers and social activities, were focussed on the present and were concerned about their other children’s well-being.^21^

Parents had difficulty understanding the disease, differed in their views regarding the aetiology and pathophysiology of the disease, especially the genetic aetiology, and thus questioned their role in their children’s illness. Parents held dichotomous views in that they wanted society to accept their children as normal, yet they needed their children to be classified as having a rare, incurable disease by social services in order to care for their children’s needs. This was compounded by the parents’ observation of the lack of knowledge of HCPs. Parents felt empowered when they received practical advice regarding the care of their babies and understood the disease when it was explained in an illustrative, simplified manner. Similar to these findings, Kahraman et al.^7^ found that parents felt that the guidance they received from HCPs was inadequate and that visual and practical education was essential. As a result, some parents sought out second opinions from other HCPs.

Parents experienced many emotions ranging from joy and excitement at the birth of their baby to fear, shock and disappointment. This is evident in the literature as well which showed that parents expressed feelings of excitement that were replaced by fear as evidenced by Wu,^19^ and anxiety, fear, frustration and depression as noted by Bruckner.^22^ There was a myriad of reasons for parents feeling guilty, from a mother questioning if she should have had a second baby knowing that she had already lost a baby with EB, to the realisation that they may have passed on the condition to their children, the latter finding is similar to the findings of Kearney et al.^23^ They felt guilty for inflicting pain during dressing changes, for not having finances to provide care for their children, and for feeling ambivalent about the life and death of their children. This was noted in other studies in which parents felt powerless, sad, stressed, helpless and distressed for inflicting pain on their children during dressing changes. It was thus recommended that parents receive physical assistance to alleviate this burden.^17,24,25^

There was an overwhelming fear of the untimely death of their children compounded by a lifespan timeframe outlined by the HCPs. Van Scheppingen noted that parents were concerned about the diminished life expectancy of their children because of the progressive nature of the disease that resulted in difficult life changes.^25^ Parents also feared having other children who may also inherit the disease. Parents of older and adult children feared their children being bullied and ostracised, whether they would make meaningful friendships and find love, and who would care for them if they, the parents, passed away.

Vulnerable parents who lack support and have poor coping skills are at a higher risk of poor self-esteem, anxiety and depression.^22^ Consistent with other studies,^19^ parents experienced suicidal ideation and resorted to self-harm to identify with their child’s pain, as well as inflicting harm on their children out of frustration.

Parents felt that HCPs were not familiar with the disease and had the unwanted burden of being the medical expert and advocate for their children, having to fight for their children to get the care that they need and deserve. This was also noted in other studies in which the parents took on the role of the expert.^19,25,26^ Parents were consumed and wrapped up in caring for their children at the expense of their well-being and relationships with their partners, family and friends.^19,25^

There is a substantial financial sacrifice and burden with parents giving up employment or studies to care for their children,^24,25,27^ salaries being docked when they take their children for medical appointments compounded by being denied financial assistance by authorities who do not understand the gravitas of the disease. Chogani et al. pointed out that parents who are of low socioeconomic status and unemployed had lower quality of life scores and were, therefore, more vulnerable and required special attention.^18^ Coping strategies differed among parents in the study, and this was influenced by the level of support from partners, having lost other children with EB and financial security. Parents were empowered by being educated about their children’s illnesses and being a part of a support group.^19^

A lack of support and judgement from family and society can have a profound effect on parents. Parents carry the guilt of passing on the disease to their children, which may be reinforced by family, compounded by traditional beliefs that they had evoked anger from the ancestors.^11^ Shizha noted that if a family did not perform certain traditional rituals, the ancestors would be angered, which may cause illness in the family.^28^ This has also been found in other cultures such as Chinese cultural bias which assigned blame to the mother for the genetic inheritance of the disease coupled with the belief that sin has led to the illness as found by Wu et al.^19^ To seek forgiveness, the parents would need to perform ceremonies that cost lots of money, which they may not have, adding to their stress and financial strife. The community in which they live and society at large add to their shame, fears, stress and anger by ignorantly accusing them of heinous acts of child abuse, being insensitive, staring and making assumptions that their children are contagious.^25^

All parents had a deep bond with their children, feared for their safety and were therefore reluctant for others to assist and care for them; the latter adding to their exhaustion and lack of downtime, similar to findings that were observed by Mauritz.^24^ The relationship between couples was arduous or solidified depending on the families’ pre-existing support structure and dynamic. Some parents banded together in their moments of anguish, whereas other couples separated, leaving the mother as the main carer, vulnerable and unsupported. Couples’ intimate relationships suffered with partners sleeping in separate rooms and seldom having quality time alone, as noted in other studies in the literature.^29^ Fine et al. reported that having a child with EB influenced a couple’s decision to have other children. Parents spent little time together outside of caring for their child with EB.^29^ Parents shared different sentiments regarding the views of their other children. Some siblings were highly supportive, having given up many social activities without resenting their sibling with EB. Other siblings were angry that they were deprived of quality time with their parents and for perceived double standards of parenting.

The role of an HCP is multifaceted and includes providing biopsychosocial support for patients and their families, including them in their care and decision-making, advocating for their care, and educating and empowering the patient and their families. Parents had contrasting experiences with the care received by HCPs. Some parents trusted their child’s HCP. They felt supported, cared for and heard, giving them comfort and empowerment to care for their children. However, many parents felt that HCPs were not experienced, some not having heard of the condition before consulting their child, causing a delay in the diagnosis of patients. They witnessed their child going through immense pain and suffering with the progression of the disease because of a lack of knowledge and care. Some HCPs accused families of abusing their children, having no understanding of the pathophysiology of the disease. They were callous in refusing a father time with his partner and child while their child was in the final hours of life. Some parents suffered at the hands of HCPs who should have been a source of comfort and support. In a study conducted by Chateau et al., HCPs shared that they did not know about the disease prior to being transferred to work in the dermatology clinic at a tertiary hospital. They admitted feeling anguish, helpless and fearful when managing a child with EB, some making excuses to leave the clinic when they knew that a child with EB was booked that day.^10^

Parents felt more supported by their GP than a specialist. They felt heard and appreciated the time spent in the consultation and the continuity of care. The GP was holistic in caring for the entire family (family unit), being available at their point of need.

Parents could be resilient when supported, empowered, and educated about their children’s illnesses. Parents have a need for specialist centres for rapid diagnosis, appropriate management and holistic care, similar to the findings by Yuen et al.^26^ Parents face emotional and physical strain and recognise a need for referral for psychological assistance, a finding concurring with Wu et al.^19^ Support groups were a source of education, comfort and support. Some parents felt that they learnt more from support groups than from the HCPs. Psychosocial guidelines by Martin et al. noted that social media and face-to-face support groups are beneficial for families of patients with EB.^17^ Parents faced financial difficulties, with many unable to provide basic care for their children. In our local setting, many families live in informal settlements without flushing ablution facilities, electricity or a communal tap that supplies many families in a community with clean water. This makes caring for a child with EB who has open wounds extremely difficult. There is a high unemployment rate among parents, with some having to give up their jobs to care for their children while others had difficulty finding employment. As a result, it is nearly impossible for parents to purchase dressings and ointments that the hospital cannot supply, and parents may default on follow-up appointments as they cannot afford the transport fare to the hospital. Many parents could not afford private medical aid, and those who did have this facility still had to pay exorbitant co-payments and continually advocate for comprehensive treatment coverage. Many parents depend on their other children’s social support grant to care for their family, which is approximately R500.00/child, an equivalent to $27.00 or £24.00, way below the breadline to adequately care for children with high needs such as EB. Some parents were despondent when they were denied financial assistance via the Care Dependency Grant.

There was a need for home-based care to assist parents and offer respite. Kearney et al. proposed that home visits would support families and prevent patients from travelling long distances for medical care.^23^ Advocacy groups and HCPs need to hold the government accountable for funding and care for patients with rare diseases. Medical aids also need to be held accountable for funding, provide home-based care and develop wound care bandage coverage programmes, as in countries such as Australia.^30^

It was interesting to note that parents felt more supported by their GP with whom they shared a close bond over the years as opposed to specialists who, at times, knew more about EB but did not offer this support. A study by Lewis supported our findings that patients prefer their GPs for the benefit of continuity of care but stressed the need for unrestricted access to a specialist if needed.^31^

### Limitations

As a rare disease, the sample was limited by availability and convenience. However, an integrated account of the parent experience was achieved within the limitations of the sample.

### Recommendations

Medical facilities:

- Need for specialist centres for comprehensive care including rapid diagnostics, appropriate management of patients, and support of the parents and family.
- Home-based care to prevent patients from travelling long distances to health facilities.

Education:

- There is a need for continuous medical education of HCPs and the development of culturally sensitive holistic biopsychosocial care guidelines for patients and their families with EB in a resource-limited environment. The authors are in the process of drafting holistic biopsychosocial care guidelines, the first to our knowledge in Africa. The multidisciplinary team will include patients, dermatologists, paediatricians, a physician, palliative care specialists, a geneticist, a dietician, a clinical psychologist, a physiotherapist, an occupational therapist, a social worker, a podiatrist, a gynaecologist, an orthopaedic surgeon and an ophthalmologist. A multidisciplinary approach of care would add to the extent to which families feel supported in addition to adding to the quality of care and maximising outcomes for the patient.
- Parents, siblings and family should be educated on the aetiology, clinical manifestations, complications, management of the conditions and the various available facilities to seek help. This aims to alleviate self-blame and empower couples.
- Communities should be educated to prevent judgement and encourage the support of patients and families afflicted with this condition.

Empower families:

- Refer families to clinical psychologists for continuous support. Identify couples at risk, and provide coping strategies.
- Siblings of patients with EB should be empowered and supported if in need.

Finances:

- Ensure that all families in financial need are referred to SASSA for a care dependency grant if the patient is a child or a disability grant if the patient is an adult.
- Parents or patients with EB who cannot work in the job market should be empowered with skills to seek alternative employment, such as working online. This should be supported by the Department of Labour.

## Conclusion

Caring for a child with an incurable, painful condition such as EB may have a negative impact on the physical, emotional, psychological, and financial well-being of the parents and siblings of patients. Healthcare practitioners should be educated about rare diseases such as EB and rapidly refer to specialist centres. There is also a need for HCPs to fully engage with parents and families, and to listen, for each patient has a meaningful story. The biopsychosocial care of parents of patients with EB within a cultural context is a crucial component of holistic family-centred care supported at the primary health level. The health authorities must prioritise rare diseases such as EB to ensure quality care for patients and their families. It is important to understand the impact a disease has on a family in order to effect change. To our knowledge, this is the first study in Africa that addresses the impact, perception and needs of a family with EB.

## Appendix 1

**TABLE 1-A1:** The questions for the in-depth interviews with the parents.

**Epidermolysis bullosa** What do you understand about the condition? What do you think is the cause of the condition?
**Emotions** What were your feelings when you saw your baby for the first time? How did the condition affect your attachment with the baby? Did the condition affect how your newborn bonded with you? Did you have difficulty feeling a mother or parental love for the baby? What are your greatest fears?
**Quality of life** How has it affected your day-to-day activities such as work or household tasks? How has having a baby with EB affected your finances and the household?
**Coping** How do you manage when you feel stressed or anxious about the baby’s condition? Who do you go to for support or advice?
**Support** What practical support do you have at home? Is your partner involved in the financial, physical and emotional support of the baby? Has your baby’s illness affected your relationship with your partner? What financial support do you have for yourself and the baby? What emotional support do you have at home? What moral or spiritual support do you have? Have you felt supported by the medical team? If so, in what ways have you felt supported?
**Needs** What assistance have you needed (or do you need) to care for the baby, if any?
**Family perceptions** What are the maternal family beliefs about the baby’s condition? What are the paternal family beliefs about the baby’s condition? What has the head of the family or other elders said about the baby’s condition and treatment?
**Parents who have lost babies/children** Can you share your journey with me? Tell me about your experience of loss How has the bereavement affected others in the family? How has the loss of the baby affected your relationship with your partner in any way? How has the loss of the baby affected your relationships with your family in any way? How are you coping now? What are your plans for the future? Have you had other babies since your baby’s death? If yes: ■ Does your baby have EB? ■ How did you feel when you found out that you were pregnant?

## References

[1] Has C, Liu L, Bolling MC, et al. Clinical practice guidelines for laboratory diagnosis of epidermolysis bullosa. Br J Dermatol. 2020;182(3):574–592. 10.1111/bjd.18128 31090061 PMC7064925

[2] Has C, Bauer JW, Bodemer C, et al. Consensus reclassification of inherited epidermolysis bullosa and other disorders with skin fragility. Br J Dermatol. 2020;183(4):614–627. 10.1111/bjd.18921 32017015

[3] Has C, Hess M, Anemüller W, et al. Epidemiology of inherited epidermolysis bullosa in Germany. J Eur Acad Dermatol Venereol. 2023;37(2):402–410. 10.1111/jdv.18637 36196047

[4] Fine J-D, Mellerio J. Extracutaneous manifestations and complications of inherited epidermolysis bullosa Part I. Epithelial associated tissues. J Am Acad Dermatol. 2009;61:367–384; quiz 85. 10.1016/j.jaad.2009.03.052 19700010

[5] Fine J-D, Mellerio J. Extracutaneous manifestations and complications of inherited epidermolysis bullosa. Part II. Other organs. J Am Acad Dermatol. 2009;61:387–402; quiz 3. 10.1016/j.jaad.2009.03.053 19700011

[6] David A, Alimohamed MZ, Modern G, et al. The plight of rare diseases in southern Africa: Health and social services policy recommendations. Qeios [Internet]. 2023 Jun 15;1.

[7] Kahraman S, Çiftçi E, Timuçin A. Determination of caregiving burden of parents providing care to their children with epidermolysis bullosa. Egypt J Dermatol Venerol. 2017;37:1. 10.4103/1110-6530.207488

[8] Ayibor, P. K. Treatment received by children who visit traditional healers (Doctoral dissertation). Faculty of Health Sciences, University of the Witwatersrand, 2008. https://hdl.handle.net/10539/7466

[9] Smith JA, Flowers P, Larkin M, Interpretative phenomenological analysis: Theory, method and research, London: Sage; 2009.

[10] Chateau AV, Aldous C, Dlova N, Blackbeard D. ‘It breaks my heart’: Healthcare practitioners’ caring for families with epidermolysis bullosa. Health SA. 2023;28:2355. 10.4102/hsag.v28i0.2355 37927945 PMC10623493

[11] Chateau AV, Gqaleni N, Aldous C, Dlova N, Blackbeard D. A qualitative study on traditional healers’ perceptions and management of epidermolysis bullosa. Health SA. 2023;28:2266. 10.4102/hsag.v28i0.2266 37670748 PMC10476505

[12] Chateau AV, Blackbeard D, Aldous C. The impact of epidermolysis bullosa on the family and healthcare practitioners: A scoping review. Int J Dermatol. 2023;62(4):459–475. 10.1111/ijd.16197 35524482

[13] Alase AO. The interpretative phenomenological analysis (IPA): A guide to a good qualitative research approach. Int J Educ Liter Stud. 2017;5:9–19. 10.7575/aiac.ijels.v.5n.2p.9

[14] Smith JA, Nizza IE. Essentials of interpretative phenomenological analysis. Washington, DC: American Psychological Association; 2022.

[15] Lincoln YS, Guba EG. But is it rigorous? Trustworthiness and authenticity in naturalistic evaluation. N Direct Prog Eval. 1986;1986(30):73–84. 10.1002/ev.1427

[16] Shenton A. Strategies for ensuring trustworthiness in qualitative research projects. Educ Inform. 2004;22:63–75. 10.3233/EFI-2004-22201

[17] Martin K, Geuens S, Asche JK, et al. Psychosocial recommendations for the care of children and adults with epidermolysis bullosa and their family: Evidence based guidelines. Orphanet J Rare Dis. 2019;14(1):133. 10.1186/s13023-019-1086-5 31186066 PMC6560722

[18] Chogani F, Parvizi MM, Murrell DF, Handjani F. Assessing the quality of life in the families of patients with epidermolysis bullosa: The mothers as main caregivers. Int J Womens Dermatol. 2021;7(5Part B):721–726. 10.1016/j.ijwd.2021.08.007 35028371 PMC8714583

[19] Wu YH, Sun FK, Lee PY. Family caregivers’ lived experiences of caring for epidermolysis bullosa patients: A phenomenological study. J Clin Nurs. 2020;29(9–10):1552–1560. 10.1111/jocn.15209 32043289

[20] Antonio da Silva R, da Silva dos Santos RE, da Silva Alencastro LC, Tomazelli Ferreira R. Coping strategies in the experience of motherhood against epidermolysis bullosa. Estrategias Enfrentamiento Vivencia Maternidad Frente Epidermólisis Bullosa. 2023;37:1–10.

[21] Cardinali P, Migliorini L, Rania N. The caregiving experiences of fathers and mothers of children with rare diseases in Italy: Challenges and social support perceptions. Front Psychol. 2019;10:1780. 10.3389/fpsyg.2019.01780 31428029 PMC6690318

[22] Bruckner AL, Losow M, Wisk J, et al. The challenges of living with and managing epidermolysis bullosa: Insights from patients and caregivers. Orphanet J Rare Dis. 2020;15(1):1. 10.1186/s13023-019-1279-y 31900176 PMC6942340

[23] Kearney S, Donohoe A, McAuliffe E. Living with epidermolysis bullosa: Daily challenges and health-care needs. Health Expect. 2020;23(2):368–376. 10.1111/hex.13006 31868299 PMC7104643

[24] Mauritz P, Jonkman MF, Visser SS, Finkenauer C, Duipmans JC, Hagedoorn M. Impact of painful wound care in epidermolysis bullosa during childhood: An interview study with adult patients and parents. Acta Dermato Venereol. 2019;99(9):783–788. 10.2340/00015555-3179 30896776

[25] Van Scheppingen C, Lettinga AT, Duipmans JC, Maathuis CG, Jonkman MF. Main problems experienced by children with epidermolysis bullosa: A qualitative study with semi-structured interviews. Acta Dermato Venereol. 2008;88(2):143–150. 10.2340/00015555-0376 18311442

[26] Yuen WY, Duipmans JC, Jonkman MF. The needs of parents with children suffering from lethal epidermolysis bullosa. Br J Dermatol. 2012;167(3):613–618. 10.1111/j.1365-2133.2012.10993.x 22512671

[27] Dufresne H, Hadj-Rabia S, Bodemer C. Impact of a rare chronic genodermatosis on family daily life: The example of epidermolysis bullosa. Br J Dermatol. 2018;179(5):1177–1178. 10.1111/bjd.16710 29710387

[28] Shizha E, Charema J. Health and wellness in Southern Africa: Incorporating indigenous and western healing practices. International Journal of Psychology and Counselling. 2011;3(9):167–75.

[29] Fine JD, Johnson LB, Weiner M, Suchindran C. Impact of inherited epidermolysis bullosa on parental interpersonal relationships, marital status and family size. Br J Dermatol. 2005;152(5):1009–1014. 10.1111/j.1365-2133.2004.06339.x 15888161

[30] Stevens LJ. Access to wound dressings for patients living with epidermolysis bullosa – An Australian perspective. Int Wound J. 2014;11(5):505–508. 10.1111/j.1742-481X.2012.01116.x 23174001 PMC7950898

[31] Lewis CL, Wickstrom GC, Kolar MM, et al. Patient preferences for care by general internists and specialists in the ambulatory setting. J Gen Intern Med. 2000;15(2):75–83. 10.1046/j.1525-1497.2000.05089.x 10672109 PMC1495342

