# Corrigendum: The impact, perceptions and needs of parents of children with epidermolysis bullosa

**DOI:** 10.4102/safp.v66i1.5993

**Published:** 2024-07-16

**Authors:** Antoinette V. Chateau, David Blackbeard, Colleen Aldous, Ncoza Dlova, Cassidy-Mae Shaw

**Affiliations:** 1 Department of Dermatology, Faculty of Health Sciences, Greys Hospital, Pietermaritzburg, South Africa; 2 Department of Dermatology, Faculty of Health Sciences, University of KwaZulu-Natal, Durban, South Africa; 3 Department of Psychology, Faculty of Health Science, Greys Hospital, Pietermaritzburg, South Africa; 4 Department of Psychiatry, Faculty of Health Science, University of KwaZulu-Natal, Durban, South Africa; 5 School of Clinical Medicine, Faculty of Health Sciences, University of KwaZulu-Natal, Durban, South Africa

In the published article, Chateau AV, Blackbeard D, Aldous C, Dlova N, Shaw C-M. The impact, perceptions and needs of parents of children with epidermolysis bullosa. S Afr Fam Pract. 2024;66(1), a5897. https://doi.org/10.4102/safp.v66i1.5897, there was an error in affiliation 4.

Instead of:

Department of Psychology, Faculty of Health Science, University of KwaZulu-Natal, Durban, South Africa

It should be:

Department of Psychiatry, Faculty of Health Science, University of KwaZulu-Natal, Durban, South Africa

The authors apologise for this error. The correction does not change the study’s findings of significance or overall interpretation of the study’s results or the scientific conclusions of the article in any way.

